# Supplementary material for: Exploring the Spatial Features of Electronic Transitions in Molecular and Biomolecular Systems by Swift Electrons
Source: J Chem Theory Comput. 2021 Mar 1;17(4):2364–73. doi: 10.1021/acs.jctc.1c00045 (PMC8047794; doi:10.1021/acs.jctc.1c00045)
Supplement: Supplementary file 1 — ct1c00045_si_001.pdf [file ct1c00045_si_001.pdf]

Exploring the spatial features of electronic transitions in molecular and biomolecular systems by swift electrons

Ciro A. Guido,*,† Enzo Rotunno,*,‡ Matteo Zanfrognini,‡ Stefano Corni,†,‡ and Vincenzo Grillo‡

†*Dipartimento di Scienze Chimiche, Università di Padova, via F. Marzolo 1, 35131 Padova, Italy.*

‡*CNR-NANO, Institute of Nanoscience, via Campi 213/A, Modena, Italy*

E-mail: ciro.guido@unipd.it; enzo.rotunno@nano.cnr.it

Table of content

- Numerical integration procedure of the OAM-EELS rate
- Computational details
- Coordinates of simulated systems

Numerical integration procedure of the OAM-EELS rate

The numerical integration of the energy loss rate per unit of angular momentum, eq(10), imply the discretization of the molecular transition potential $V_{0n}(\mathbf{r})$. Due to the dimension of the large systems treated, we use a linear response TD-DFT approach:[1] indeed, the optimal compromise between accuracy and computational cost makes TD-DFT the most widely used method of calculating excitation energies of chemically relevant systems.

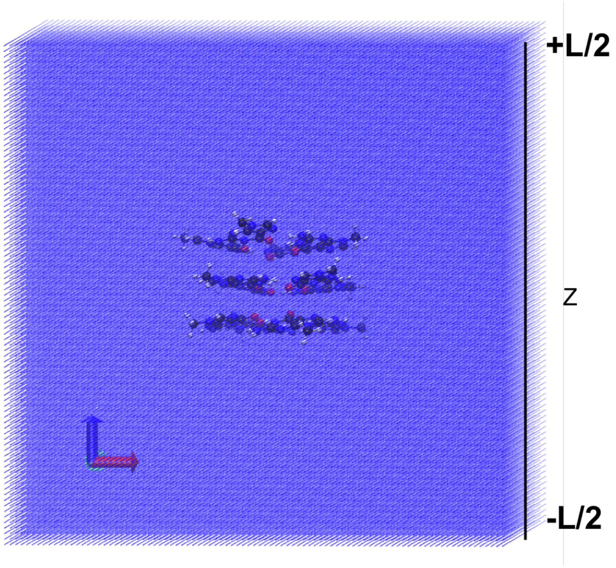

Figure 1: cube grid centerd on a G-quadruplex

The transition potential is therefore expressed as:

$$V_{0n}(\mathbf{r}) = \int d\mathbf{r}' \frac{\rho(\mathbf{r}', \omega_{0n})}{|\mathbf{r} - \mathbf{r}'|} = \sum_{ia} \int d\mathbf{r}' \frac{[\phi_i(\mathbf{r}')\phi_a^*(\mathbf{r}')X_{ia}(\omega_{0n}) + \phi_a(\mathbf{r}')\phi_i^*(\mathbf{r}')Y_{ia}(\omega_{0n})]}{|\mathbf{r} - \mathbf{r}'|} \tag{1}$$

$X_{ia}(\omega_{0n})$ and $Y_{ia}(\omega_{0n})$ refer to the excitation and de-excitation coefficients involving all the possible pair of occupied $\phi_i(\mathbf{r}')$ and virtual $\phi_a(\mathbf{r}')$ orbitals that describe a transition from the ground ($|0\rangle$) to an excited state ($|n\rangle$) associated to an energy difference of ω_{0n}. The quantity in eq.(1) have been discretized over a cubic grid (with a length side of L, fig.1) by slightly modifying the input of the G16 software,[2] and then averaged along the direction $p = k_{z,f} - k_{z,i}$:

$$\widetilde{V}_p^{0n}(\mathbf{r}_\perp) = V_{0n}(x, y, p) = \frac{1}{L} \sum_{z=-L/2}^{L/2} V_{0n}(x, y, z) \cdot e^{-ipz} \tag{2}$$

The eq.(2) of the manuscript have been finally integrated over the grid cube, by a home-made Matlab script.

Computational details

We report in this section the computational details used in the simulations, the geometrical structures, the excitation energies, the oscillator strengths and the final OAM resolved transition probabilities of studied systems.

Coordinates of the structures

The coordinates (in Angstrom) of the geometrical structures of different molecular systems are here reported.

Table 1: Coordinates (Angstrom) of the optimized guanine structure.

Guanine optimized			
Atomic num.	X	Y	Z
6	0.527396	-0.848145	-0.000496
6	0.852889	0.503229	0.006425
7	-0.694082	-1.431941	0.006852
6	-1.669670	-0.564036	-0.003188
7	2.220761	0.676588	0.006069
1	3.755825	-0.784696	0.000844
6	2.708797	-0.530077	0.001573
7	1.726856	-1.501188	-0.002774
1	1.849904	-2.499212	-0.010476
6	-0.209959	1.468808	0.002677
8	-0.191592	2.680114	-0.002454
7	-1.473623	0.787371	-0.003150
1	-2.265157	1.410920	-0.066178
7	-2.968795	-1.004832	-0.066863
1	-3.064106	-1.992225	0.107193
1	-3.678270	-0.436363	0.365378

Table 2: Coordinates (Angstrom) of the guanine tetramer. The first 16 atoms correspond to the extracted monomer.

Guanine tetramer (first part)				Guanine tetramer (second part)			
Atomic num.	X	Y	Z	Atomic num.	X	Y	Z
6	7.124327	2.025491	3.630807	6	0.907212	7.201075	3.638859
6	7.815970	3.240532	3.585003	6	2.114835	6.498413	3.586990
7	5.779680	1.840050	3.646899	7	0.733688	8.546110	3.681540
6	5.078949	2.987362	3.615867	6	1.885702	9.237920	3.663245
7	9.188858	3.018085	3.572269	7	1.873441	5.130012	3.528787
1	10.253991	1.158362	3.607276	1	0.001022	4.086986	3.507830
6	9.312348	1.696283	3.612156	6	0.548714	5.021713	3.548995
7	8.092717	1.044264	3.654290	7	-0.086233	6.246998	3.624083
1	7.897188	0.019295	3.665785	1	-1.108426	6.463969	3.646210
6	7.074620	4.463291	3.546078	6	3.345340	7.229355	3.570226
8	7.505263	5.628678	3.473075	8	4.505915	6.784180	3.499096
7	5.672352	4.231785	3.580682	7	3.122776	8.633953	3.624554
1	5.093909	5.099009	3.547453	1	3.976361	9.228723	3.590084
7	3.726185	2.927002	3.630253	7	1.824653	10.590499	3.692353
1	3.304059	2.017258	3.485154	1	0.910106	11.009906	3.570142
1	3.117740	3.762059	3.543164	1	2.633870	11.229254	3.625942
6	5.852214	13.716316	3.680861	6	12.061521	8.545953	3.682014
6	5.161402	12.500891	3.644500	6	10.855591	9.247327	3.595163
7	7.196996	13.903079	3.682239	7	12.235376	7.200783	3.718500
6	7.898841	12.756290	3.643754	6	11.085274	6.507889	3.658726
7	3.788280	12.723387	3.631236	7	11.097535	10.616488	3.550845
1	2.722884	14.582529	3.654934	1	12.968740	11.661071	3.593525
6	3.664574	14.045178	3.664321	6	12.420692	10.725698	3.613989
7	4.884248	14.697496	3.700946	7	13.053965	9.501126	3.704474
1	5.078446	15.722432	3.693632	1	14.075958	9.283000	3.713083
6	5.902649	11.279515	3.587592	6	9.628760	8.515255	3.519822
8	5.472693	10.113603	3.507215	8	8.471900	8.960287	3.399092
7	7.305081	11.511765	3.608361	7	9.849819	7.110408	3.574685
1	7.882460	10.646625	3.542254	1	8.996743	6.515313	3.528476
7	9.250935	12.817400	3.651762	7	11.145335	5.155953	3.681858
1	9.672655	13.729362	3.519903	1	12.066682	4.735256	3.660888
1	9.860913	11.983377	3.563293	1	10.340430	4.513925	3.596018

Table 3: Coordinates (Angstrom) of the L-alanine.

L-alanine			
Atomic num.	X	Y	Z
7	1.280452	-1.135863	0.238015
1	-0.363503	-1.588108	-0.623873
6	0.621780	0.167930	0.423392
1	0.672247	0.513619	1.458338
6	1.249350	1.229605	-0.479824
6	-0.876223	0.046556	0.088166
1	1.230769	0.907089	-1.522457
1	0.696031	2.162401	-0.398987
1	2.285385	1.413109	-0.191154
8	-1.201375	-1.079117	-0.561303
1	1.324027	-1.662055	1.101125
8	-1.673970	0.900814	0.364784
1	2.225200	-1.033135	-0.107347

Table 4: Coordinates (Angstrom) of the D-alanine.

D-alanine			
Atomic num.	X	Y	Z
6	0.621864	-0.167998	0.423331
6	1.249342	-1.229479	-0.480095
6	-0.876217	-0.046528	0.088182
1	0.696051	-2.162307	-0.399261
1	1.230606	-0.906912	-1.522707
1	2.285426	-1.413035	-0.191631
8	-1.201354	1.078865	-0.561779
1	0.672170	-0.513967	1.458206
7	1.280547	1.135824	0.238280
1	-0.363487	1.587840	-0.624691
1	1.322755	1.662345	1.101268
8	-1.674042	-0.900579	0.365257
1	2.225884	1.033002	-0.105468

Table 5: Coordinates (Angstrom) of Gquadruplex 2MB2. First layer.

2MB2 - first layer (first part)				2MB2 - first layer (second part)			
Atomic num.	X	Y	Z	Atomic num.	X	Y	Z
6	-3.254354	-2.520009	3.027107	6	-1.145159	5.399237	-3.732626
1	-3.615623	-1.951693	2.156515	1	-0.883116	6.054751	-4.577057
1	-3.696043	-2.090807	3.939316	1	-0.958827	5.940084	-2.792310
1	-3.559108	-3.571993	2.931273	1	-2.208457	5.126829	-3.795233
7	-1.804667	-2.462697	3.096973	7	-0.352438	4.182943	-3.777142
6	-0.905280	-3.510106	3.055813	6	-0.794386	2.878406	-3.879705
1	-1.248047	-4.541619	2.959861	1	-1.859524	2.649050	-3.939624
7	0.366387	-3.127127	3.143061	7	0.187854	1.980425	-3.895731
6	0.279688	-1.753096	3.246367	6	1.328116	2.752663	-3.797745
6	1.327627	-0.760867	3.371064	6	2.720950	2.356271	-3.764128
8	2.543734	-0.867965	3.417564	8	3.252149	1.257151	-3.812210
7	0.724964	0.542113	3.445551	7	3.535398	3.535682	-3.653803
1	1.409075	1.291761	3.534907	1	4.532526	3.328660	-3.624804
6	-0.617204	0.845832	3.408707	6	3.109093	4.842947	-3.587898
7	-0.951457	2.168681	3.496852	7	4.078294	5.801862	-3.485174
1	-0.261798	2.899210	3.584967	1	5.061783	5.580365	-3.457685
1	-1.933988	2.403151	3.470828	1	3.776681	6.765003	-3.436089
7	-1.574554	-0.053387	3.295354	7	1.842902	5.208748	-3.618109
6	-1.054936	-1.317645	3.220142	6	1.019261	4.120342	-3.722976
6	-3.097976	1.497085	-0.309350	6	1.731854	7.899708	3.080344
1	-3.153362	2.121525	-1.214025	1	2.237432	8.214789	2.154849
1	-3.237622	2.138749	0.573936	1	2.258830	8.340305	3.940343
1	-3.890598	0.735784	-0.339369	1	0.690353	8.251641	3.068085
7	-1.812428	0.824310	-0.242286	7	1.729559	6.450787	3.182228
6	-1.558860	-0.533163	-0.217259	6	0.643040	5.601175	3.254976
1	-2.374154	-1.257747	-0.248700	1	-0.376382	5.990101	3.239851
7	-0.264054	-0.834513	-0.153336	7	0.974387	4.315033	3.341418
6	0.350980	0.401445	-0.136259	6	2.354694	4.338981	3.323193
6	1.757867	0.740471	-0.073224	6	3.305706	3.248312	3.388467
8	2.758335	0.041394	-0.019341	8	3.148301	2.039912	3.475302
7	1.889913	2.171910	-0.080791	7	4.636076	3.790257	3.334426
1	2.859539	2.481690	-0.038081	1	5.359018	3.073612	3.376141
6	0.878924	3.104494	-0.137420	6	4.995682	5.115474	3.236956
7	1.253639	4.419403	-0.132428	7	6.334903	5.388730	3.201697
1	2.218471	4.709824	-0.089758	1	7.038459	4.667420	3.243972
1	0.519643	5.112632	-0.173698	1	6.610308	6.358299	3.130238
7	-0.402710	2.801058	-0.194895	7	4.134131	6.111531	3.177260
6	-0.587297	1.444622	-0.190527	6	2.845980	5.650675	3.225095

Table 6: **Coordinates (Angstrom) of Gquadruplex 2MB2. Second layer.**

2MB2 - second layer (first part)				2MB2 - second layer (second part)			
Atomic num.	X	Y	Z	Atomic num.	X	Y	Z
6	6.403772	7.893023	-0.415215	6	12.125116	2.886545	3.266823
1	7.049051	7.916503	-1.306557	1	12.527775	2.364080	2.385731
1	7.024049	8.069236	0.476768	1	12.505761	2.395505	4.175348
1	5.640577	8.680523	-0.493360	1	12.451516	3.936300	3.252405
7	5.734173	6.608008	-0.314547	7	10.673102	2.853114	3.249549
6	4.377395	6.349556	-0.311433	6	9.794306	3.918243	3.220037
1	3.650859	7.159820	-0.391015	1	10.158334	4.946841	3.206453
7	4.079223	5.056819	-0.204897	7	8.513829	3.555320	3.210659
6	5.316615	4.448388	-0.135441	6	8.572641	2.176164	3.235600
6	5.659057	3.046467	-0.010572	6	7.503677	1.198647	3.239615
8	4.962462	2.045699	0.065373	8	6.288776	1.326194	3.222433
7	7.090739	2.920590	0.020281	7	8.080312	-0.117822	3.269826
1	7.402871	1.954696	0.107381	1	7.380402	-0.858139	3.274216
6	8.020803	3.932803	-0.053371	6	9.417375	-0.444607	3.292011
7	9.336555	3.564400	-0.003372	7	9.724982	-1.776546	3.319182
1	9.629314	2.603209	0.083053	1	9.019908	-2.497602	3.323171
1	10.027954	4.299351	-0.056624	1	10.703529	-2.027934	3.335633
7	7.714254	5.209935	-0.167048	7	10.393787	0.441154	3.288467
6	6.357444	5.388526	-0.201366	6	9.899475	1.717419	3.259976
6	9.799810	5.400193	-3.625789	6	12.203566	-1.681881	0.115429
1	10.425291	5.101525	-4.480774	1	12.277459	-2.362728	-0.746206
1	10.356925	5.211727	-2.695450	1	12.294398	-2.269988	1.041338
1	9.561856	6.470917	-3.700994	1	13.015105	-0.941606	0.069332
7	8.556826	4.648695	-3.627000	7	10.933217	-0.978053	0.090951
6	7.265767	5.132977	-3.706332	6	10.712108	0.383589	0.022403
1	7.070956	6.204120	-3.779910	1	11.545453	1.086640	-0.021970
7	6.335145	4.181749	-3.683252	7	9.423560	0.716816	0.017324
6	7.070775	3.017706	-3.583230	6	8.778327	-0.501796	0.086662
6	6.628718	1.639958	-3.515114	6	7.362210	-0.805120	0.115818
8	5.511512	1.145742	-3.527389	8	6.377792	-0.082156	0.087637
7	7.782425	0.787842	-3.418337	7	7.195632	-2.230971	0.191371
1	7.542766	-0.201015	-3.366476	1	6.217813	-2.516025	0.215426
6	9.104503	1.170460	-3.391328	6	8.184961	-3.187332	0.231613
7	10.032517	0.171079	-3.294103	7	7.778332	-4.490777	0.303092
1	9.778816	-0.803682	-3.243650	1	6.805776	-4.756619	0.326099
1	11.006166	0.440524	-3.273451	1	8.496215	-5.201241	0.333149
7	9.511822	2.422757	-3.453030	7	9.474874	-2.916411	0.205335
6	8.449485	3.281055	-3.546690	6	9.692230	-1.566756	0.133254

Table 7: Coordinates (Angstrom) of Gquadruplex 2MB2. Third layer.

2MB2 - third layer (first part)				2MB2 - third layer (second part)			
Atomic num.	X	Y	Z	Atomic num.	X	Y	Z
6	10.264334	-5.881469	-2.844563	6	2.674049	-7.963378	-0.010491
1	10.039485	-6.645291	-3.604461	1	2.047804	-8.037974	-0.912533
1	10.001309	-6.279392	-1.852644	1	2.035104	-8.090235	0.876687
1	11.336835	-5.641151	-2.871385	1	3.439105	-8.752751	-0.028118
7	9.511502	-4.668916	-3.114232	7	3.340750	-6.673621	0.031913
6	9.996512	-3.400940	-3.368503	6	4.697034	-6.413138	0.049148
1	11.069435	-3.202881	-3.386490	1	5.425480	-7.225379	0.030594
7	9.043636	-2.495722	-3.578340	7	4.992262	-5.115963	0.089132
6	7.877692	-3.224188	-3.451595	6	3.753385	-4.506656	0.098136
6	6.497612	-2.800442	-3.570178	6	3.407703	-3.100507	0.136754
8	6.002500	-1.709366	-3.808858	8	4.102054	-2.095893	0.171026
7	5.644143	-3.936350	-3.351521	7	1.975629	-2.975485	0.130241
1	4.653617	-3.708249	-3.420750	1	1.661260	-2.006746	0.156306
6	6.027484	-5.228869	-3.073201	6	1.047819	-3.991788	0.093971
7	5.026490	-6.144683	-2.903731	7	-0.268874	-3.623355	0.095386
1	4.050083	-5.902732	-2.975545	1	-0.563859	-2.659316	0.121451
1	5.296447	-7.096645	-2.699013	1	-0.958636	-4.361289	0.068937
7	7.281901	-5.619432	-2.964288	7	1.357319	-5.272776	0.058752
6	8.141568	-4.572987	-3.164123	6	2.714638	-5.450748	0.063188
6	7.189287	-7.496018	3.367719	6	-0.724050	-5.752736	-3.318720
1	6.674313	-7.908501	2.486762	1	-1.341779	-5.485040	-4.189453
1	6.713007	-7.894807	4.276319	1	-1.281003	-5.511132	-2.400647
1	8.247620	-7.793068	3.347109	1	-0.503448	-6.829549	-3.341921
7	7.115512	-6.045422	3.358216	7	0.531091	-5.022423	-3.350711
6	8.155711	-5.137253	3.328774	6	1.814283	-5.530824	-3.401724
1	9.193943	-5.472507	3.308891	1	1.991736	-6.607277	-3.423455
7	7.757368	-3.867315	3.327670	7	2.760302	-4.594806	-3.420214
6	6.380498	-3.964523	3.358185	6	2.043545	-3.415534	-3.378661
6	5.373728	-2.923146	3.371985	6	2.507918	-2.043461	-3.374425
8	5.467446	-1.705099	3.360639	8	3.633114	-1.568589	-3.405728
7	4.073910	-3.536257	3.404783	7	1.368037	-1.169089	-3.322943
1	3.314487	-2.857202	3.416007	1	1.623714	-0.182903	-3.317245
6	3.784444	-4.881974	3.421339	6	0.039780	-1.528478	-3.283056
7	2.461683	-5.226578	3.452576	7	-0.872026	-0.510669	-3.237231
1	1.721369	-4.541825	3.463344	1	-0.602536	0.461142	-3.232213
1	2.237614	-6.211798	3.464960	1	-1.850025	-0.762986	-3.207684
7	4.696926	-5.833386	3.408888	7	-0.387823	-2.775517	-3.286718
6	5.958849	-5.303689	3.377550	6	0.660590	-3.654431	-3.335158

Table 8: **Coordinates (Angstrom) of Gquadruplex 143D. First layer**

143D - first layer (first part)				143D - first layer (second part)			
Atomic num.	X	Y	Z	Atomic num.	X	Y	Z
6	11.972350	-1.982580	4.206760	6	8.050410	-6.764150	-3.986640
1	12.110290	-2.882020	3.587570	1	7.715160	-6.940330	-5.020080
1	11.883200	-2.287380	5.260600	1	7.453850	-7.392570	-3.307950
1	12.840590	-1.318550	4.088600	1	9.113150	-7.030380	-3.894760
7	10.780530	-1.264630	3.789800	7	7.892910	-5.362340	-3.640410
6	10.684360	0.029460	3.316790	6	8.867420	-4.463020	-3.254130
1	11.570800	0.657440	3.213920	1	9.912530	-4.766620	-3.174800
7	9.441270	0.398520	3.016880	7	8.399850	-3.242460	-3.003090
6	8.694380	-0.723880	3.314430	6	7.045470	-3.363980	-3.241910
6	7.268100	-0.949990	3.201200	6	5.990240	-2.376670	-3.142170
8	6.360470	-0.226120	2.820710	8	6.011760	-1.196470	-2.827160
7	6.975480	-2.288220	3.637120	7	4.742070	-2.995730	-3.496200
1	5.983850	-2.515690	3.583370	1	3.952310	-2.353980	-3.447230
6	7.867000	-3.232510	4.093770	6	4.534650	-4.303330	-3.873250
7	7.348210	-4.445070	4.454320	7	3.248150	-4.663100	-4.164630
1	6.363510	-4.654640	4.395150	1	2.476930	-4.015460	-4.111280
1	7.993640	-5.145840	4.791010	1	3.084300	-5.620660	-4.442570
7	9.165690	-3.031610	4.198640	7	5.491490	-5.205500	-3.965500
6	9.501630	-1.767430	3.794920	6	6.707100	-4.667890	-3.637960
6	10.250340	-6.200190	1.457250	6	-2.507820	-3.932410	-3.542700
1	10.646130	-5.730080	2.370360	1	-2.827990	-3.703550	-4.570580
1	11.005460	-6.119500	0.660560	1	-3.159380	-3.391900	-2.839320
1	10.035240	-7.260350	1.653500	1	-2.591630	-5.013970	-3.364040
7	9.019280	-5.545970	1.049530	7	-1.123610	-3.537240	-3.348870
6	7.762010	-6.100890	0.911700	6	-0.049090	-4.331880	-3.000140
1	7.589200	-7.158710	1.116310	1	-0.173580	-5.402710	-2.831210
7	6.833740	-5.231840	0.518690	7	1.098850	-3.665790	-2.900310
6	7.535070	-4.048880	0.396650	6	0.741650	-2.366860	-3.202510
6	7.078450	-2.732410	0.000860	6	1.552830	-1.168290	-3.262990
8	5.975030	-2.321270	-0.324670	8	2.744920	-0.985780	-3.067660
7	8.194860	-1.827240	0.029310	7	0.713060	-0.058630	-3.623490
1	7.943210	-0.877420	-0.239900	1	1.222310	0.821650	-3.684470
6	9.497210	-2.116410	0.368870	6	-0.639560	-0.081930	-3.878210
7	10.389900	-1.081890	0.320850	7	-1.224900	1.109770	-4.204780
1	10.124730	-0.146720	0.052140	1	-0.706490	1.973010	-4.260940
1	11.348810	-1.282810	0.568130	1	-2.217500	1.104370	-4.393810
7	9.917650	-3.312650	0.729680	7	-1.382070	-1.169940	-3.824490
6	8.890910	-4.218030	0.720110	6	-0.629960	-2.261900	-3.483950

Table 9: Coordinates (Angstrom) of Gquadruplex 143D. Second layer

143D - second layer (first part)				143D - second layer (second part)			
Atomic num.	X	Y	Z	Atomic num.	X	Y	Z
6	-1.232110	-5.128430	0.776320	6	6.023280	7.614230	3.168110
1	-0.947920	-5.516940	1.766130	1	6.787090	7.724880	2.383400
1	-1.027060	-5.900890	0.019560	1	6.526550	7.516710	4.142090
1	-2.304160	-4.884660	0.771310	1	5.374530	8.501800	3.177060
7	-0.481660	-3.921380	0.476970	7	5.203000	6.444060	2.908080
6	-0.968190	-2.654430	0.220500	6	3.838940	6.386210	2.699150
1	-2.040590	-2.452820	0.224770	1	3.229060	7.290910	2.715890
7	-0.017380	-1.754770	-0.020360	7	3.382110	5.154480	2.485680
6	1.148760	-2.485950	0.087540	6	4.518870	4.374660	2.561790
6	2.527160	-2.068280	-0.066740	6	4.676710	2.942190	2.415610
8	3.020280	-0.981480	-0.328000	8	3.866070	2.056300	2.190660
7	3.381680	-3.204820	0.144390	7	6.065420	2.610090	2.582040
1	4.371110	-2.980910	0.050330	1	6.248930	1.612080	2.491100
6	3.000550	-4.493030	0.444850	6	7.106590	3.473360	2.838470
7	4.002160	-5.410520	0.601160	7	8.350740	2.918890	2.957150
1	4.977460	-5.172740	0.504690	1	8.515840	1.928330	2.864360
1	3.733820	-6.359360	0.821900	1	9.122510	3.543350	3.145400
7	1.747670	-4.878060	0.586260	7	6.968110	4.777530	2.972320
6	0.887100	-3.830760	0.395030	6	5.659560	5.150750	2.822640
6	2.236320	-7.612410	5.801350	6	10.993390	5.273920	-0.636590
1	1.703930	-8.005770	4.921990	1	10.621550	5.896970	0.191040
1	1.498860	-7.353610	6.576330	1	10.780040	5.783860	-1.588370
1	2.922550	-8.379090	6.188650	1	12.078520	5.132230	-0.530940
7	3.011520	-6.439900	5.435220	7	10.351900	3.971010	-0.610910
6	4.376780	-6.254200	5.531260	6	10.943490	2.729900	-0.479330
1	5.024740	-7.040640	5.921610	1	12.025360	2.631160	-0.376690
7	4.785110	-5.059250	5.110810	7	10.076750	1.719940	-0.492330
6	3.613940	-4.440330	4.721640	6	8.856430	2.348340	-0.640960
6	3.397910	-3.117140	4.173240	6	7.523890	1.786460	-0.723930
8	4.174920	-2.211180	3.912410	8	7.127550	0.631470	-0.685820
7	1.992190	-2.940870	3.929490	7	6.578180	2.858340	-0.876300
1	1.767950	-2.026100	3.541080	1	5.615160	2.532260	-0.941340
6	0.983150	-3.847950	4.161990	6	6.846080	4.207200	-0.938170
7	-0.286600	-3.449960	3.847700	7	5.773130	5.041530	-1.086770
1	-0.492080	-2.540370	3.463650	1	4.825330	4.702740	-1.149660
1	-1.035170	-4.107910	4.014280	1	5.958160	6.033810	-1.133370
7	1.174600	-5.053130	4.660830	7	8.056820	4.723390	-0.863150
6	2.501600	-5.275560	4.913010	6	9.000900	3.742940	-0.716740

Table 10: **Coordinates (Angstrom) of Gquadruplex 143D. Third layer.**

143D - third layer (first part)				143D - third layer (second part)			
Atomic num.	X	Y	Z	Atomic num.	X	Y	Z
6	11.967630	3.831900	-4.250140	6	-0.391950	6.167290	-0.418760
1	12.424150	3.196200	-5.024020	1	-0.762450	6.005690	0.604980
1	12.378670	3.547780	-3.269450	1	-1.157520	5.824770	-1.131510
1	12.202270	4.886220	-4.455320	1	-0.197980	7.238200	-0.574470
7	10.524230	3.669610	-4.255040	7	0.847110	5.437840	-0.624670
6	9.557110	4.631330	-4.472820	6	2.089060	5.940820	-0.959630
1	9.831060	5.668340	-4.673520	1	2.240940	7.011470	-1.105740
7	8.312970	4.162580	-4.411140	7	3.030580	5.007380	-1.076590
6	8.490410	2.820970	-4.137710	6	2.354540	3.835630	-0.800700
6	7.509910	1.770910	-3.952710	6	2.834720	2.469410	-0.767600
8	6.288820	1.786990	-3.987590	8	3.941950	1.994400	-0.970020
7	8.197730	0.536760	-3.687580	7	1.739820	1.601970	-0.428120
1	7.564380	-0.248710	-3.546610	1	2.008760	0.620160	-0.387010
6	9.557670	0.337010	-3.612300	6	0.436750	1.962520	-0.169220
7	9.978850	-0.936270	-3.346240	7	-0.432790	0.951860	0.133920
1	9.338700	-1.703400	-3.209120	1	-0.150650	-0.015670	0.170530
1	10.975200	-1.094400	-3.288730	1	-1.392000	1.205100	0.325770
7	10.453890	1.289200	-3.779870	7	-0.005090	3.204260	-0.198060
6	9.851540	2.491440	-4.036390	6	1.000480	4.076420	-0.517720
6	0.642570	7.050140	-4.597350	6	-3.802880	1.798490	3.877630
1	1.011050	7.309980	-5.601400	1	-3.999530	2.359630	2.951420
1	1.165650	7.672330	-3.855260	1	-3.671620	2.513030	4.704460
1	-0.438440	7.242580	-4.541570	1	-4.654430	1.137850	4.094730
7	0.876640	5.642540	-4.325970	7	-2.610910	0.982400	3.726090
6	-0.052640	4.661520	-4.040120	6	-2.497260	-0.390530	3.824960
1	-1.117980	4.893070	-3.993850	1	-3.367370	-1.012200	4.042120
7	0.482790	3.459880	-3.838170	7	-1.259390	-0.840160	3.632760
6	1.835480	3.681100	-4.003840	6	-0.534640	0.310950	3.396470
6	2.948050	2.758180	-3.910830	6	0.876470	0.481890	3.117560
8	2.990450	1.561950	-3.666350	8	1.786710	-0.326880	3.018070
7	4.167170	3.475350	-4.167590	7	1.147710	1.882930	2.944230
1	4.994590	2.882990	-4.117920	1	2.127790	2.076070	2.744000
6	4.304450	4.814040	-4.457540	6	0.250320	2.924000	3.020990
7	5.576100	5.271510	-4.665060	7	0.747600	4.181340	2.817950
1	6.385220	4.671840	-4.612260	1	1.721080	4.355890	2.620580
1	5.688630	6.252700	-4.878640	1	0.097690	4.953170	2.871980
7	3.295240	5.657900	-4.543300	7	-1.034620	2.773620	3.274500
6	2.104380	5.025550	-4.306640	6	-1.349860	1.452950	3.449030

Optimized Guanine

The geometrical structure have been optimized using the B3LYP excahnge-correlation (xc) functional and the cc-pVTZ basis set. TD-DFT simulations have been done using the CAM-B3LYP xc-functional and the aug-cc-pVTZ basis set. The cubic grid, centered on the molecular structure, have a spacing of 0.1 a.u., a side of 40 a.u., and 68921 grid points. The annular shaped electron beam have the following dimensions: 7 a.u. of radius and 3 a.u. thick.

Table 11: Excitation energy (eV), oscillator strength and OAM resolved transition rates of the optimized guanine structure.

	Guanine		Orbital angular momentum						
Transition	energy	Osc. Str.	-3	-2	-1	0	1	2	3
$S_1 \leftarrow S_0$	5.056	0.133	0.0	0.7	16.0	0.0	16.0	0.7	0.0
$S_2 \leftarrow S_0$	5.167	0.023	0.0	0.2	2.5	0.0	2.5	0.2	0.0
$S_3 \leftarrow S_0$	5.514	0.079	0.1	0.5	8.4	0.0	8.4	0.5	0.1
$S_4 \leftarrow S_0$	5.553	0.000	0.0	0.0	0.0	0.0	0.0	0.0	0.0
$S_5 \leftarrow S_0$	5.557	0.244	0.2	1.2	26.7	0.0	26.7	1.2	0.2

Guanine tetramer

The geometry of the flat guanine tetramer has been obtained optimizing a monolayer of guanine on top of a gold slab with four layers of Au(111), as detailed in ref. 3. TD-DFT simulations have been done using the CAM-B3LYP xc-functional and the 6-311G(d) basis set. The cubic grid, centered on the molecular structure, have a spacing of 0.1 a.u., a side of 50 a.u. and 132651 grid points. The annular shaped electron beam have the following dimensions: 20 a.u. of radius and 3 a.u. thick. In the case of the monomer extracted from the structure of the tetramer, we used the same computational protocol of the total system including the grid cube: therefore this last was centered on the total structure.

Table 12: Excitation energy (eV), oscillator strength and OAM resolved transition rates of the guanine monomer extracted from the tetramer.

	Guanine Monomer		Orbital angular momentum						
Transition	energy	Osc. Str.	-3	-2	-1	0	1	2	3
$S_1 \leftarrow S_0$	5.057	0.163	0.0	0.6	19.9	0.0	19.9	0.6	0.0
$S_2 \leftarrow S_0$	5.358	0.001	0.0	0.0	0.1	0.0	0.1	0.0	0.0
$S_3 \leftarrow S_0$	5.507	0.290	0.2	1.5	32.5	0.0	32.5	1.5	0.2
$S_4 \leftarrow S_0$	5.710	0.008	0.1	0.8	0.0	0.0	0.8	0.1	0.0
$S_5 \leftarrow S_0$	6.233	0.002	0.0	0.0	0.0	0.0	0.0	0.0	0.0

Table 13: Excitation energy (eV), oscillator strength and OAM resolved transition rates of the guanine tetramer structure.

	Guanine tetramer		Orbital angular momentum								
Transition	energy	Osc. Str.	-4	-3	-2	-1	0	1	2	3	4
$S_1 \leftarrow S_0$	5.061	0.002	0.0	0.0	4.7	0.1	0.0	0.1	4.7	0.0	0.0
$S_2 \leftarrow S_0$	5.074	0.271	0.0	0.2	0.0	12.1	0.0	12.1	0.0	0.2	0.0
$S_3 \leftarrow S_0$	5.086	0.306	0.0	0.2	0.0	13.6	0.0	13.6	0.0	0.2	0.0
$S_4 \leftarrow S_0$	5.097	0.002	0.0	0.0	0.1	0.1	0.2	0.1	0.1	0.0	0.0
$S_5 \leftarrow S_0$	5.357	0.000	1.5	0.0	0.3	0.0	0.4	0.0	0.3	0.0	1.5
$S_6 \leftarrow S_0$	5.378	0.721	0.0	2.8	0.0	30.3	0.0	30.3	0.0	2.8	0.0
$S_7 \leftarrow S_0$	5.394	0.721	0.0	3.6	0.0	30.2	0.0	30.2	0.0	3.6	0.0
$S_8 \leftarrow S_0$	5.417	0.000	0.1	0.0	22.3	0.0	0.0	0.0	22.3	0.0	0.1

Alanine enantiomers

The geometrical structure have been optimized using the B3LYP excahnge-correlation (xc) functional and the cc-pVTZ basis set. TD-DFT simulations have been done using the CAM-B3LYP xc-functional and the cc-pVTZ basis set. The cubic grid, centered on the molecular structure, have a spacing of 0.1 a.u., a side of 80 a.u. and 531441 grid points. The annular shaped electron beam have the following dimensions: 7 a.u. of radius and 3 a.u. thick.

G-quadruplex structures

The geometries of the guanine core of parallel and anti-parallel G-quadruplexes have been extracted from the NMR structures of PDB files: PDB ID 2MB2[4] and 143D[5] , respectively. Therefore, they have been refined by projecting the MP2/cc-pVDZ optimized geometry of

Table 14: Excitation energy (eV), oscillator strength and OAM resolved transition rates of the L-alanine.

	L-alanine		Orbital angular momentum						
Transition	energy	Osc. Str.	-3	-2	-1	0	1	2	3
$S_1 \leftarrow S_0$	5.7623	0.0013	0.02	0.31	1.71	0.96	1.70	0.31	0.02
$S_2 \leftarrow S_0$	7.0424	0.0154	0.89	3.23	30.59	3.55	29.89	3.18	0.88
$S_3 \leftarrow S_0$	7.9932	0.0227	1.54	1.26	33.99	9.68	33.79	1.28	1.53
$S_4 \leftarrow S_0$	8.0665	0.0032	0.15	0.73	4.33	0.39	4.14	0.74	0.15
$S_5 \leftarrow S_0$	8.4068	0.0087	0.68	2.05	10.09	0.6	10.4	2.08	0.69

Table 15: Excitation energy (eV), oscillator strength and OAM resolved transition rates of the D-alanine.

	D-alanine		Orbital angular momentum						
Transition	energy	Osc. Str.	-3	-2	-1	0	1	2	3
$S_1 \leftarrow S_0$	5.7623	0.0013	0.02	0.33	1.64	1.08	1.65	0.33	0.02
$S_2 \leftarrow S_0$	7.0424	0.0154	0.76	3.25	30.01	3.33	30.7	3.3	0.77
$S_3 \leftarrow S_0$	7.9932	0.0227	1.61	1.57	34.2	8.5	34.37	1.55	1.61
$S_4 \leftarrow S_0$	8.0665	0.032	0.17	0.7	4.15	0.35	4.34	0.69	0.17
$S_5 \leftarrow S_0$	8.4068	0.0087	0.64	2.08	10.58	0.64	10.25	2.04	0.64

the guanine base to the NMR structure, as detailed in ref. 6.

All the TD-DFT calculations have been done employing the CAM-B3LYP xc-functional and the 6-31G(d) basis set. The cubic grid, centered on each supramolecular structure, have a spacing of 0.1 a.u., a side of 85 a.u. and 753571 grid points for both the structures. The annular shaped electron beam have the following dimensions: 24 a.u. of radius and 3 a.u. thick.

Table 16: Excitation energy (eV), oscillator strength and dichroic figure of merit (eq. (13) of main text) of the 2MB2 G-quadruplex.

	2MB2		Dichroic figure of merit		
Transition	energy	Osc. Str.	$\|l\| = 1$	$\|l\| = 2$	$\|l\| = 3$
$S_1 \leftarrow S_0$	4.938	0.004	5.9	0.7	2.2
$S_2 \leftarrow S_0$	5.064	0.0208	0.8	1.3	2.8
$S_3 \leftarrow S_0$	5.065	0.0001	-0.9	-0.3	-2.3
$S_4 \leftarrow S_0$	5.090	0.0007	2.2	-0.4	2.2
$S_5 \leftarrow S_0$	5.107	0.618	1.8	4.9	1.5
$S_6 \leftarrow S_0$	5.137	0.0305	2.0	-0.7	5.2
$S_7 \leftarrow S_0$	5.143	0.0152	0.1	2.8	1.7
$S_8 \leftarrow S_0$	5.151	0.0356	3.1	2.4	2.8
$S_9 \leftarrow S_0$	5.153	0.0187	1.7	-2.1	3.7
$S_{10} \leftarrow S_0$	5.166	0.0338	2.6	4.2	-7.2
$S_{11} \leftarrow S_0$	5.174	0.0946	0.5	-3.9	-0.8
$S_{12} \leftarrow S_0$	5.175	0.0241	1.8	-0.2	0.7
$S_{13} \leftarrow S_0$	5.181	0.0328	-0.1	-0.4	-0.2
$S_{14} \leftarrow S_0$	5.195	0.0909	0.8	-2.6	-0.6
$S_{15} \leftarrow S_0$	5.207	0.0162	-1.2	9.1	-0.7

Table 17: Excitation energy (eV), oscillator strength and dichroic figure of merit (eq. (13) of main text) of the 143D G-quadruplex.

	143D		Dichroic figure of merit		
Transition	energy	Osc. Str.	$\|l\| = 1$	$\|l\| = 2$	$\|l\| = 3$
$S_1 \leftarrow S_0$	4.9987	0.1197	1.5	1.3	2.1
$S_2 \leftarrow S_0$	5.0264	0.0315	3.0	7.9	-2.9
$S_3 \leftarrow S_0$	5.0938	0.0398	0.8	0.9	3.4
$S_4 \leftarrow S_0$	5.1059	0.0386	-1.1	1.0	-1.0
$S_5 \leftarrow S_0$	5.1232	0.0438	0.4	-1.8	-2.8
$S_6 \leftarrow S_0$	5.1259	0.0390	0.7	-0.2	0.5
$S_7 \leftarrow S_0$	5.1443	0.0400	-0.8	-0.6	-2.3
$S_8 \leftarrow S_0$	5.1715	0.0182	7.7	-2.4	1.4
$S_9 \leftarrow S_0$	5.1839	0.0444	-0.7	-4.1	2.5
$S_{10} \leftarrow S_0$	5.1922	0.0155	7.4	2.9	-1.8
$S_{11} \leftarrow S_0$	5.1988	0.0118	-4.4	-4.6	-1.2
$S_{12} \leftarrow S_0$	5.2147	0.0544	0.4	0.0	-1.2
$S_{13} \leftarrow S_0$	5.2287	0.0308	-1.1	0.5	-1.9
$S_{14} \leftarrow S_0$	5.2333	0.2754	-1.0	0.1	-2.0
$S_{15} \leftarrow S_0$	5.2459	0.1652	0.2	-2.7	-2.1

References

(1) Casida, M. E. In *Time-Dependent Density-Functional Response Theory for Molecules*; Chong, D. P., Ed.; Recent Advances in Density Functional Methods; World Scientific: Singapore, 1995; Vol. 1; pp 155–192.

(2) Frisch, M. J.; Trucks, G. W.; Schlegel, H. B.; Scuseria, G. E.; Robb, M. A.; Cheeseman, J. R.; Scalmani, G.; Barone, V.; Petersson, G. A.; Nakatsuji, H. et al. Gaussian˜16 Revision A.03. 2016; Gaussian Inc. Wallingford CT.

(3) Rosa, M.; Corni, S.; Di Felice, R. Enthalpy–Entropy Tuning in the Adsorption of Nucleobases at the Au(111) Surface. *J. Chem. Theory Comput.* **2014**, *10*, 1707–1716.

(4) Sengar, A.; Heddi, B.; Phan, A. T. Formation of G-Quadruplexes in Poly-G Sequences: Structure of a Propeller-Type Parallel-Stranded G-Quadruplex Formed by a G15 Stretch. *Biochemistry* **2014**, *53*, 7718–7723.

(5) Wang, Y.; Patel, D. J. *Structure* **1993**, *1*, 263–282.

(6) Jurinovich, S.; Cupellini, L.; Guido, C. A.; Mennucci, B. EXAT: EXcitonic analysis tool. *J. Comp. Chem.* **2018**, *39*, 279–286.
